# How Long Does Adaption Last for? An Update on the Psychological Impact of the Confinement in Portugal

**DOI:** 10.3390/ijerph19042243

**Published:** 2022-02-16

**Authors:** Ana Daniela Costa, Afonso Fernandes, Sónia Ferreira, Beatriz Couto, Mafalda Machado-Sousa, Pedro Moreira, Pedro Morgado, Maria Picó-Pérez

**Affiliations:** 1 Psychological Neuroscience Lab, CIPsi, School of Psychology, University of Minho, 4710-057 Braga, Portugal; adanieladcosta@gmail.com (A.D.C.); pedromsmoreira@gmail.com (P.M.); 2 Life and Health Sciences Research Institute (ICVS), School of Medicine, University of Minho, 4710-057 Braga, Portugal; a81199@alunos.uminho.pt (A.F.); soniamgaf@gmail.com (S.F.); beatriz.azevedo.couto@gmail.com (B.C.); mafaldagmsousa@gmail.com (M.M.-S.); mariapico231@gmail.com (M.P.-P.); 3 ICVS/3B’s, PT Government Associate Laboratory, 4710-057 Guimarães, Braga, Portugal

**Keywords:** COVID-19, mental health, confinement, adaptation, DASS-21, Portugal

## Abstract

During the first COVID-19 related confinement in Portugal, there was a decrease in the levels of psychological symptoms measured by the Depression, Anxiety and Stress Scale 21 (March to April 2020). Upon experiencing a new period of restraints in 2021, the psychological impact of this sample was assessed again (*N* = 322, two more time points). It was expected that the psychological symptoms evidenced in February 2021 would be at similar levels to those found in April 2020, leading to a transfer of adaptation. Contrary to our hypothesis, in the second confinement in Portugal there were higher levels of depression and stress symptoms than at the beginning of the pandemic. On the other hand, the maximum level of anxiety was observed in March 2020. It seems that our perception of the threats in 2021 was not the same as at the onset of COVID-19, or that knowledge was not disseminated to the general population to increase their mental health literacy and help them cope with the imposed challenges.

## 1. Introduction

Since December 2019, the world has heard about a new disease caused by the virus SARS-CoV-2. By March 2020, the World Health Organization (WHO) declared it a pandemic and humans around the globe have been living in unprecedented times ever since [1].

From the experience with past epidemics, harmful effects of confinement on a psychological level were expected (i.e., increased levels of anxiety, mood and stress symptoms) and have been well documented since the outbreak of COVID-19, e.g., Refs. [2,3].

In Portugal, immediate moderate to severe levels of depression, anxiety and stress were found in about 12–15%, 11–16%, and 6–17% of the population, respectively [4,5].

During the first contact with new and contagious diseases that lead to the imposition of restrictive measures, people live emerged in fear [6,7]. In the presence of an imminent threat of death, people become hypervigilant and experience an altered sense of security [8,9]. Indeed, the anticipation of contracting a disease seems to wield the same impact on mental health as actually being infected [10]. The levels of psychological symptoms seem to decrease alongside the increase in information about the pathogenic agent (i.e., transmission pathways, treatment options, and the number of cases and deaths in the living area), and the adoption of precautionary measures (e.g., handwashing, use of mask, …) [11].

As one of the idiosyncrasies of this pandemic is its pervasiveness and the long-term reliance on public health measures to contain it, studies characterizing its waves and the emergence of new strains of the virus are lacking. In fact, literature reporting COVID-19 long-term psychological impact is scarce, having the longitudinal studies published only covered a few months after the pandemic commencement [12,13,14].

In Portugal, two distinct periods of mandatory confinement due to COVID-19 were instituted, in March 2020 and January 2021. Taking this into account, we aimed to explore the psychological impact (i.e., levels of depression, anxiety, and stress) of the second mandatory confinement in Portugal. The mental health impact of this new period of restrictions will be compared to the initial response of the same sample. An adaptation is expected [12,15,16] as people had already faced an analogous experience, and preparedness and coping strategies are thought to be perceived as superior [17]. Thus, lower levels of depressive, anxiogenic, and stress symptoms are anticipated in comparison to March of 2020.

## 2. Materials and Methods

From a Portuguese sample of 2040 adult subjects (detailed methods described in [5], [15]) we analysed 322 participants that responded to the Depression, Anxiety and Stress Scale (DASS-21) [18,19] at four time points (two in 2020 and two in 2021).

The subsample (*N* = 322, 81.1% female) had a mean age of 41.44 (*SD* = 12.75) years old and 17.96 (*SD* = 3.43) mean education years.

The DASS-21 assesses the levels of self-reported psychological symptomatology in the previous week, within a 4-point Likert scale ranging from “Did not apply to me at all” (0) to “Applied to me very much, or most of the time” (3). It is divided in three subscales, each of them with seven items: Depression (Cronback’s α = 0.85), Anxiety (Cronback’s α = 0.74) and Stress (Cronback’s α = 0.81). In this study, the DASS-21 was applied through an online survey in Google Forms and was used as a repetitive measure of the psychological impact of COVID-19.

The first data collection took place in March of 2020, days after the beginning of the first mandatory confinement in Portugal. One month after, the participants responded again to the online questionnaire. At the beginning of 2021, a new period of mandatory confinement was declared, which led to the acquisition of data in two more time points as follows: February and March. In February of 2021, about one month had elapsed since the newly imposed restrictions.

Due to the non-normality of the data, Friedman tests, non-parametric analysis of repeated measures, were performed to the DASS-21 subscales to analyse differences in the levels of symptomatology throughout time. Post hoc analyses followed to understand the specific time points driving the statistical differences. All statistical analyses were performed with the Statistical Package for the Social Sciences, version 27 (SPSS 27).

All the study procedures here described followed the ethical requirements for human research in agreement with the Declaration of Helsinki, and were accordingly approved by the Ethical Committee for Life Sciences of the University of Minho.

## 3. Results

### 3.1. Evolution of the Psychological Symptoms and the Epidemiological Context in Portugal

**Figure 1:**
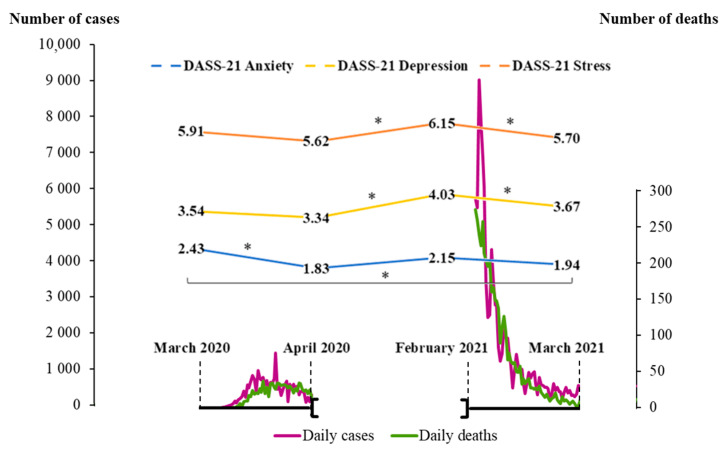
Evolution of the COVID-19 pandemic in Portugal: number of infected cases (magenta line), number of deaths (green line) and psychological impact (mean scores on DASS-21). * *p* < 0.008.

Figure 1 illustrates the mean scores obtained in DASS-21 in the four different time points, as well as the evolution of the pandemic in Portugal in the corresponding moments.

The higher mean score for the DASS-21 subscales of depression and stress was registered in February 2021, whereas for anxiety it was observed in March of 2020. In February of 2021, a mean of 3002 new daily cases of infection by COVID-19 and 137 mean daily deaths were registered. This contrasts with the numbers reached in March of 2020, the mean number of daily cases being 240, and the mean number of deaths being 5.

### 3.2. Severity of the Psychological Symptoms

Considering the severity classification of the DASS-21 scores of the sample (Table 1), the majority of the participants presented normal ranges of psychological symptoms in all subscales and time points. Moreover, the percentage of participants within moderate to severe levels of symptomatology was within 14–19% in the stress subscale, 14–18% in the depression subscale and 7–12% in the anxiety subscale.

### 3.3. DASS-21: A Comparison of Confinements

Regarding the evolution of the symptoms throughout time, there were statistically significant differences regarding the DASS-21 subscales of stress, χ2(3) = 11.350, *p* = 0.010, Kendall’s W = 0.012, depression, χ2(3) = 20.701, *p* < 0.001, Kendall’s W = 0.021, and anxiety, χ2(3) = 28.403, *p* < 0.001, Kendall’s W = 0.029.

Post-hoc analysis with a Bonferroni correction revealed that the levels of stress and depression were statistically different in February 2021 compared to April 2020 (Z stress = −2.721, *p* = 0.007; Z depression = −2.208, *p* < 0.001), and to March 2021 (Z stress = −2.579, *p* = 0.010; Z depression = −2.949, *p* = 0.003). Thus, there was a significant increase in stress and depressive symptoms from April 2020 to February 2021, followed by a significant decrease from the latter to March 2021. However, regarding anxiety, the scores were statistically different in March 2020 compared to April 2020 (Z = −4.479, *p* < 0.001), and March 2021 (Z = −3.233, *p* = 0.001). The evolution of the anxiogenic symptoms demonstrates a peak in the first evaluation, levels that were not statistically different to the ones at the beginning of the second confinement (February 2021), and were significantly superior to the other two time points.

## 4. Discussion

Although the experience of a second confinement does not convey novelty, people exhibit distress. Even more, it seems that facing it for a second time leads to poorer outcomes than the first time. In Portugal, during the second period of pandemic restrictions, there was an increase in symptomatology as measured by DASS-21, in comparison to the last evaluation in 2020. The levels of stress and depressive symptomatology reached in February 2021 were even superior to the ones obtained at the onset of the outbreak emergence. Thus, the adaptation developed during the first confinement [5,15] was not automatically transferable when re-living such restrictions.

The results contradict our hypothesis that people would have learned how to positively cope with measures of physical distancing in that one year. The improved knowledge about the disease and how to ensure our protection may explain why the anxiogenic levels had a spike at the beginning of the pandemic in Portugal and not in February of 2021. On the other hand, the pervasiveness of public health measures, without any guarantee of a rapid return to our old paths, highlight the enhanced feelings of hopelessness and strain that the idea of a second confinement may pose [20,21,22].

In 2021, the epidemiological scenario was different from 2020, as follows: whereas in March of 2020, few cases and deaths were described, in February of 2021, Portugal had just faced the worst month of reports. However, our appraisal of the impact of the pandemic is not based solely on the number of cases and deaths. The weight of the appearance of a new case at the beginning of COVID-19 may have been comparable to the burden of hundreds or thousands of cases nowadays.

The uncertainty and misinformation spread, at the start, made COVID-19 an extremely relevant threat [23], wiring us all to the news and updates [24,25]. Coupled with a threatening climate of economic instability due to the global shutdown of several industries and consequent lay-offs or unemployment, most people’s reaction was displayed in the form of anxiety. However, nowadays the threat perception seems to be guided by different emotions [26]. We hypothesized that there was a shift of attention to the losses (i.e., death of loved ones, liberty, time, etc.) suffered throughout this entire time and to the signs of exhaustion, resulting in a widespread fatigue, a feeling of defeat and powerlessness in response to the unfolding of the COVID-19 pandemic.

The psychological responses presented here are normative, and for most of us, time-restricted [3]. Studies like this are important to characterize the idiosyncrasies experienced in each phase of the COVID-19 pandemic [27] and inform us on how can we thrive in the possibility of new confinement [28].

As the psychological responses fluctuated, the strategies used previously to mitigate them, such as keeping healthy routines and being socially connected, are no longer being effective. In response to such a continuous stressor, the singularities and complexity of each one’s response to the pandemic should be addressed. Therefore, mental health professionals should be playing a pivotal role in the management of the pandemic [29] and the development of psychological interventions within primary health care [23]. The harmful consequences of this pandemic will prevail long before its acute phases, similar to what happened with Severe Acute Respiratory Syndrome (SARS) [20], influenza A (H1N1) [30] and Middle East Respiratory Syndrome (MERS) [31], so their mitigation should be a priority and a matter of public concern [23,32,33]. Improvements in mental health literacy would help us overcome the stigma associated with seeking help, and timely assessment and intervention could promote resilience [9,34] and prevent the shifting to long-lasting patterns of malfunctioning [30,33,35,36].

The limitations of the present study are worthy of note and must be considered when interpreting the results. The subsample analysed has an overrepresentation of females and of highly educated backgrounds, which limits the generalization of results to the general Portuguese population. In fact, when considering gender, Connor and colleagues [37] highlighted the additional burden that may be associated with being female in the current pandemic. Furthermore, the complexity of human’s response to events, especially as stressful and unprecedented as the current, should be understood in light of a biopsychosocial framework [26,38]. Thus, some hypotheses were formulated to interpret the results, but the mechanisms underlying the psychological impact described remain to be completely portraited and may be of multifactorial origin (e.g., Refs. [5,39,40]).

## 5. Conclusions

The psychological adaptation seen during the first confinement in Portugal was not observed in the first moment of the second confinement on national territory. This may have happened because the threats were perceived differently in 2021 and/or because the general population is not equipped to deal with such stressful events. Investments should be made in mental health services (i.e., literacy and universal access in primary care) in order to prevent the cumulative effect of this pandemic from becoming damaging and for lessons to be learned from the experience with COVID-19, thereby allowing for post-traumatic growth.

## Figures and Tables

**Table 1:** Subsample distribution according to the severity of psychological symptoms and time.

DASS-21 Subscales	Severity Levels	March 2020 (%)	April 2020 (%)	February 2021 (%)	March 2021 (%)
Stress	Normal	70.8	72.7	70.5	74.5
	Mild	13.4	10.6	9.0	9.6
	Moderate	7.8	8.7	9.3	6.8
	Severe	7.1	5.0	9.6	6.8
	Extremely Severe	0.9	3.1	1.6	2.2
Depression	Normal	69.6	71.7	65.2	69.3
	Mild	13.4	11.2	13.7	9.9
	Moderate	12.1	11.5	12.1	13.0
	Severe	3.2	2.8	5.3	5.0
	Extremely Severe	1.9	2.8	3.7	2.8
Anxiety	Normal	74.5	82.3	79.8	78.9
	Mild	10.2	7.1	7.8	11.2
	Moderate	7.8	5.3	4.7	4.3
	Severe	3.7	3.1	2.8	3.1
	Extremely Severe	3.7	2.2	5.0	2.5

## Data Availability

The data is available at https://osf.io/3rztv/ (accessed on 16 January 2022).
